# A nomogram based on CT texture features to predict the response of patients with advanced pancreatic cancer treated with chemotherapy

**DOI:** 10.1186/s12876-023-02902-4

**Published:** 2023-08-10

**Authors:** Jingjing Li, Jiadi Du, Yuying Li, Mingzhu Meng, Junjie Hang, Haifeng Shi

**Affiliations:** 1 Graduate College, Dalian Medical University, Dalian, China; 2 Department of Radiology, Changzhou Second People’s Hospital, Changzhou, China; 3 Department of Computer Science, Missouri University of Science and Technology, Rolla, MO U.S.; 4 Department of Medical Oncology, National Cancer Center, National Clinical Research Center for Cancer/Cancer Hospital & Shenzhen Hospital, Chinese Academy of Medical Sciences, Peking Union Medical College, 518116 Shenzhen, China; 5 Department of Oncology, the Affiliated Changzhou Second People’s Hospital of Nanjing Medical University, Changzhou Medical Center, Changzhou, China

**Keywords:** CT texture features, Advanced pancreatic cancer, Treatment response, Nomogram, Radiomics signature

## Abstract

**Objective:**

This study aimed to evaluate the predictive value of computed tomography (CT) texture features in the treatment response of patients with advanced pancreatic cancer (APC) receiving palliative chemotherapy.

**Methods:**

This study enrolled 84 patients with APC treated with first-line chemotherapy and conducted texture analysis on primary pancreatic tumors. 59 patients and 25 were randomly assigned to the training and validation cohorts at a ratio of 7:3. The treatment response to chemotherapy was evaluated according to the Response Evaluation Criteria in Solid Tumors (RECIST1.1). The patients were divided into progressive and non-progressive groups. The least absolute shrinkage selection operator (LASSO) was applied for feature selection in the training cohort and a radiomics signature (RS) was calculated. A nomogram was developed based on a multivariate logistic regression model incorporating the RS and carbohydrate antigen 19-9 (CA19-9), and was internally validated using the C-index and calibration plot. We performed the decision curve analysis (DCA) and clinical impact curve analysis to reflect the clinical utility of the nomogram. The nomogram was further externally confirmed in the validation cohort.

**Results:**

The multivariate logistic regression analysis indicated that the RS and CA19-9 were independent predictors (P < 0.05), and a trend was found for chemotherapy between progressive and non-progressive groups. The nomogram incorporating RS, CA19-9 and chemotherapy showed favorable discriminative ability in the training (C-index = 0.802) and validation (C-index = 0.920) cohorts. The nomogram demonstrated favorable clinical utility.

**Conclusion:**

The RS of significant texture features was significantly associated with the early treatment effect of patients with APC treated with chemotherapy. Based on the RS, CA19-9 and chemotherapy, the nomogram provided a promising way to predict chemotherapeutic effects for APC patients.

## Introduction

Pancreatic cancer is the seventh leading cause of cancer death, and its incidence and mortality have been stable. It has been predicted that pancreatic cancer will exceed breast cancer as the third leading cause of cancer death by 2025 [1]. The 5-year survival rates of pancreatic cancers in Nordics countries is 6% [2]. Unfortunately, about 80–85%of patients with pancreatic cancer are found to be advanced or metastatic which precludes curative resection [3]. Presently, most patients with advanced pancreatic cancer are treated with chemotherapy. The first-line chemotherapy includes FOLFIRINOX (oxaliplatin, irinotecan, fluorouracil, leucovorin), gemcitabine alone or combined with albumin-bound nab-paclitaxel [4, 5]. Despite significant progress in chemotherapy regimen, patients with APC respond differently to it. Previous studies showed that patients present poor responses to gemcitabine, which can be caused by the low expression of human equilibrative nucleoside transporter 1 (hENT1). However, the expression of hENT1 is not a routine detection in clinical practice and a large propotion of patients with APC even not received gemcitabine as first-line chemotherapy [6]. Among these problems, how to accurately evaluate the efficacy of chemotherapy at an early stage is still a challenging problem for clinicians when making clinical treatment decisions. Thus, there is an urgent need to find potential biomarkers to identify patients who can benefit from chemotherapy.

CT, compared with other effective methods like endoscopic ultrasound, is a noninvasive method in the diagnosis and treatment effect evaluation of pancreatic cancer [7]. However, conventional CT cannot quantitatively analyze tumor heterogeneity or predict therapeutic effects [8]. Fortunately, with the development of artificial intelligence, radiomics has played an important role in extracting quantitative features in medical images, which can be investigated to predict the efficacy and prognosis in targeted therapies, immunotherapies and radiotherapy [9]. Texture analysis is composed of various mathematical techniques, which can describe the grey-level patterns of images and play an important role in evaluating the spatial organization of different tissues and organs [10].

Several studies have shown that texture analysis can be used to predict treatment response in pancreatic cancer. For example, Nasief et al. found delta radiomics can be used as a biomarker for early prediction of treatment response in neoadjuvant chemoradiation therapy [11]. Simpson et al. found radiomics features may contain predictive information about response to treatment for PDAC patients undergoing stereotactic body radiotherapy (SBRT) [12]. Yue et al. found the predictive value of combining clinical features with PET-CT texture features in patients undergoing radiotherapy [13]. Nasief et al. found that combining delta-radiomics features and CA 19-9 levels results in an earlier prediction of good and bad responders undergoing neoadjuvant chemoradiation therapy [14]. To date, whether there is a correlation between CT texture features and first-line chemotherapy efficacy in patients with APC remains to be elucidated. Thus, this study aimed to determine the predictive value of pre-treatment CT texture features in APC patients receiving first-line chemotherapy.

## Materials and methods

### Patients

We retrospectively analyzed CT images of APC patients treated at Changzhou Second People’s Hospital Affiliated to Nanjing Medical University, between September 2016 and June 2019. They were randomly divided into a training cohort with 59 patients and a validation cohort with 25 patients. The inclusion criteria were as follows: (1) newly diagnosed and pathologically confirmed pancreatic adenocarcinoma; (2) absence of concurrent cancers at other sites; (3) TNM stage III or IV according to the 8th edition of the TNM staging system; (4) no prior history of radiotherapy, chemotherapy, or other treatments; (5) complete CT imaging data before chemotherapy and after two cycles of chemotherapy; (6) complete baseline clinicopathological features, including the patients’ age, sex, chemotherapy regimen, treatment effect ,tumor location, ECOG PS, TNM stage, CA19-9. The exclusion criteria were as follows: (1) failure to complete the prescribed chemotherapy regimen as scheduled; (2) incomplete clinical data at baseline; (3) poor image quality. Demographic and clinicopathologic features were collected from the electronic medical records. Informed consent was obtained from each patient, and ethical approval was accepted by the Ethics committees of Changzhou Second People’s Hospital Affiliated to Nanjing Medical University.

### Treatment response assessment

The short-term therapeutic response was evaluated based on the follow-up CT imaging before the third cycle of chemotherapy using the Response Evaluation Criteria in Solid Tumors (RECIST 1.1) [15]. In our study, the patients were divided into two groups: with progressive disease (PD) and without progressive disease (SD or PR or CR).

### CT image acquisition

Contrast-enhanced CT examinations were performed using a 128-row dual-source CT scanner (SOMATOM Definition Flash, Siemens, Germany) at 120 kV, tube current modulation, and 1 mm reconstructed section thickness. All patients were instructed to fast for at least 8 h before administering intravenous contrast (Iohexol, 1.5mL per kilogram of body weight, at a rate of 3 ml/s). After the injection of contrast agent, patients were subjected to double-helical scanning during the arterial and portal venous phases. The region of interest (ROI) was selected in primary pancreatic tumors during the arterial phase.

### Image processing

ROIs were drawn on each slice of the primary pancreatic cancer using the software Labelme (version 3.11.2, http://labelme.csail.mit.edu). Then, ROIs were extracted for texture analysis using Local Image Features Extraction (LIFEx, version 5.10, https://www.lifexsoft.org/). In the segmented tumors, the volume of interest (VOI) and histogram were calculated as first-order features. For calculations of second and high-order texture features, the number of grey levels used to resample the ROI content was set to 64.0. The Cartesian coordinates for spatial resampling were 2.0 mm (X-direction), 2.0 mm (Y-direction), and 1.0 mm (Z-direction). Texture features were evaluated using four texture matrices, including the grey-level co-occurrence matrix (GLCM), the grey-level run length matrix (GLRLM), the neighborhood grey-level different matrix (NGLDM), and the grey-level zone length matrix (GLZLM). We used the texture features of the largest cross-section of each tumor to predict therapy response.

### Statistical analysis

Statistical analysis was conducted using R software (version 3.6.1, Institute for Statistics and Mathematics, Vienna, Austria) and SPSS statistical software (version 21.0, SPSS Inc, IBM, Armonk, NY, U.S.A.). The Chi-square test and independent samples t-test were used to investigate the differences between categorical variables and continuous variables. The correlations between texture variables were assessed using Pearson’s correlation coefficient with the R package “psych”. The LASSO was applied for feature selection, and RS was calculated by summing the selected features weighted by their β-coefficients, where RS = 3.37022285 * GLZLM_LZLGE + -0.04245328 * GLZLM_LGZE + 1.25470569 * Energy. The cut-off value of RS was determined according to the Youden’s J statistics (J = sensitivity + specificity − 1). Univariate analysis and multivariate logistic regression analysis were applied to investigate independent predictive factors. A nomogram was developed using the R package “rms” to predict the treatment effect, and the discrimination power of the nomogram was evaluated by calculating the C-index. In addition, calibration plot was generated using bootstrapping with 1000 resamples. To evaluate the clinical utility of the nomogram, DCA was performed by quantifying the net benefits at different threshold probabilities, and the clinical impact curve was performed by quantifying the number of high risk at each threshold probability.

## Results

### Patients’ characteristics

The baseline clinicopathological characteristics of patients with APC in the training and validation cohorts were shown in Table 1. All the variables, including age, gender, Eastern Cooperative Oncology Group performance status (ECOG PS), chemotherapy, effect, TNM stage, primary tumor location and CA19-9, were comparable between the training and validation cohorts.

**Table 1:** Baseline clinicopathological characteristics of patients with APC

Characteristics	Training cohort (n = 59)	Validation cohort (n = 25)	P-value
Age (median, SD)	66 (9.5)	70 (11.3)	0.625
Gender			
Male	35 (59.3%)	12 (48.0%)	0.339
Female	24 (40.7%)	13 (52.0%)	
ECOG PS			
0–1	13 (22.0%)	6 (24.0%)	0.844
2	46 (78.0%)	19 (76.0%)	
Chemotherapy			
Monotherapy	27 (45.8%)	10 (40.0%)	0.627
Combination therapy	32 (54.2%)	15 (60.0%)	
Effect			
PR	10 (16.9%)	4 (16.0%)	0.911
SD	23 (39.0%)	11 (44.0%)	
PD	26 (44.1%)	10 (40.0%)	
TNM stage			
III	10 (16.9%)	7 (28.0%)	0.249
IV	49 (83.1%)	18 (72.0%)	
Primary tumor location			
Head and neck	31 (52.5%)	18 (72.0%)	0.098
Body and tail	28 (47.5%)	7 (28.0%)	
CA19-9 (U/ml)			
< 1000	40 (67.8%)	18 (72.0%)	0.703
≥ 1000	19 (32.2%)	7 (28.0%)	

### Correlations between texture parameters and treatment effect

The mean values and standard deviations of all texture features in the training cohort are shown in Table 2, and the values of these features were normalized for further analysis. There were no significant differences in GLCM parameters between the two groups with independent samples t-tests (P > 0.1, Table 2). In the histogram analysis, the progressive group showed a higher level of energy than the non-progressive group (P = 0.059). In addition, in the GLRLM analysis, the progressive group showed lower levels of LGRE (P = 0.028), SRLGE (P = 0.029), and LRLGE (P = 0.025), but higher levels of GLNU (P = 0.070) compared with the non-progressive group. In the NGLDM analysis, only Coarseness (P = 0.076) differed between the progressive and non-progressive groups. Moreover, in the GLZLM analysis, the progressive group had lower values of SZE (P = 0.064), LGZE (P = 0.019), SZLGE (P = 0.022), LZLGE (P = 0.020) and had greater levels of LZHGE (P = 0.062), GLNU (P = 0.067) than the non-progressive group.

**Table 2:** Texture features after normalization in the training cohort

Texture parameter	SD + PR (n = 33)		PD (n = 26)		P-value
	Mean	SD	Mean	SD	
Histogram					
Skewness	0.602	0.084	0.571	0.088	0.178
Kurtosis	0.053	0.049	0.073	0.069	0.199
Entropy	0.555	0.138	0.489	0.167	0.104
Energy	0.138	0.070	0.184	0.110	0.059*
GLCM					
Homogeneity	0.489	0.113	0.529	0.134	0.219
Energy	0.023	0.010	0.024	0.011	0.623
Contrast	0.070	0.047	0.062	0.048	0.482
Correlation	0.745	0.126	0.720	0.140	0.482
Entropy	0.454	0.199	0.441	0.174	0.781
Dissimilarity	0.226	0.078	0.206	0.085	0.371
GLRLM					
SRE	0.956	0.019	0.947	0.022	0.120
LRE	0.615	0.051	0.637	0.059	0.136
LGRE	0.042	0.032	0.027	0.013	0.028**
HGRE	0.323	0.131	0.356	0.129	0.337
SRLGE	0.042	0.032	0.027	0.013	0.029**
SRHGE	0.366	0.150	0.398	0.141	0.400
LRLGE	0.045	0.032	0.029	0.013	0.025**
LRHGE	0.189	0.076	0.219	0.090	0.169
GLNU	0.077	0.072	0.152	0.218	0.070*
RLNU	0.169	0.158	0.238	0.225	0.167
RP	0.941	0.025	0.930	0.029	0.124
NGLDM					
Coarseness	0.179	0.123	0.129	0.078	0.076*
Contrast	0.001	0.001	0.001	0.001	0.674
Busyness	0.176	0.117	0.213	0.127	0.250
GLZLM					
SZE	0.850	0.052	0.822	0.061	0.064*
LZE	0.101	0.042	0.117	0.055	0.208
LGZE	0.047	0.035	0.029	0.016	0.019**
HGZE	0.344	0.139	0.377	0.135	0.359
SZLGE	0.045	0.035	0.028	0.016	0.022**
SZHGE	0.396	0.165	0.417	0.146	0.600
LZLGE	0.060	0.038	0.041	0.018	0.020**
LZHGE	0.028	0.014	0.040	0.032	0.062*
GLNU	0.101	0.084	0.180	0.223	0.067*
ZLNU	0.194	0.163	0.245	0.194	0.273
ZP	0.263	0.027	0.252	0.031	0.162

**Fig. 1:**
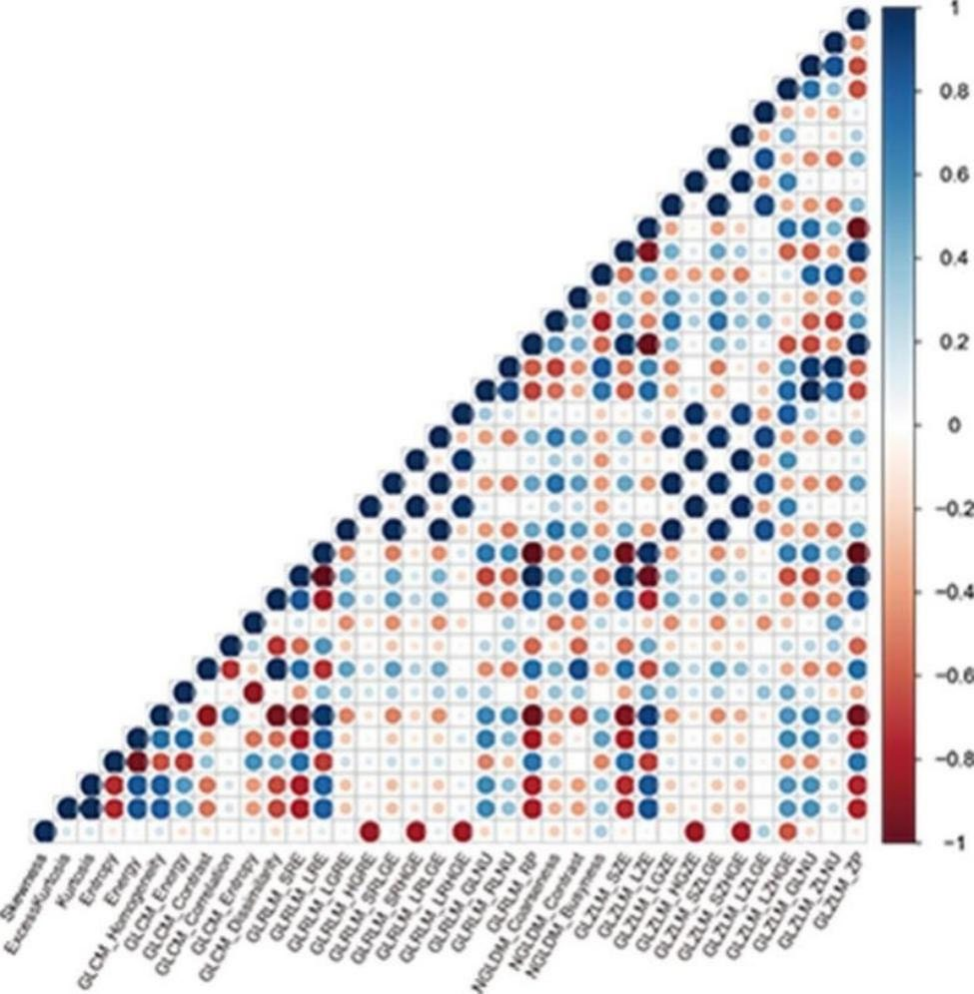
The correlations between texture parameters. The blue circles represent a positive correlation, and red circles represent a negative correlation. The darker the color of the circle is, the higher the correlation between the two texture parameters is

Pearson’s correlation was used to investigate the correlations between texture parameters in the training cohort (Fig. 1). The results indicated that some pairs of these parameters showed significant correlations.

### Texture features selection and radiomic score

**Fig. 2:**
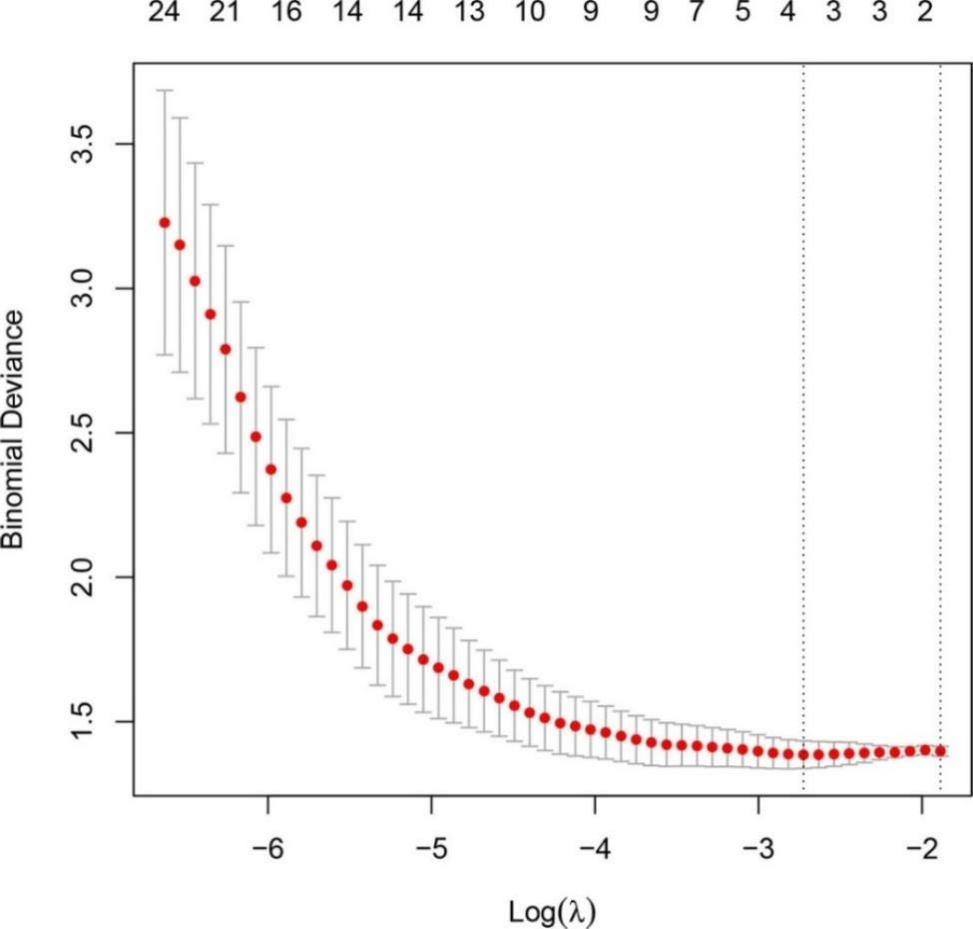
Selection of textural features using the LASSO regression. Red dots show average deviance values for each model at the given λ, and the vertical bars through the red dots indicate the upper and lower values of the binomial deviance. The vertical black lines show the optimal values of λ, where the model provides its best fit to the data

Based on the LASSO regression model, three texture features related to the treatment effect were selected (Fig. 2). The final composition of the RS is: RS = 3.37022285 * GLZLM_LZLGE + -0.04245328 * GLZLM_LGZE + 1.25470569 * Energy. In this formula, each variable was weighted using its β-coefficient derived from the LASSO model. As shown in Table 3, the RS was an independent predictor in the multivariate logistic regression analysis (P = 0.044).

**Table 3:** Comparison between clinicopathological characteristics of APC according to treatment effect in the training cohort

Characteristics	SD + PR (n = 33)	PD (n = 26)	Univariate analysis			Multivariate analysis		
			OR	95%CI	P-value	OR	95%CI	P-value
Age (median, SD)	66 (9.29)	66.5 (9.8)	1.018	0.963–1.076	0.521			
Gender								
Male	20 (60.6%)	15 (57.7%)	0.886	0.312–2.521	0.821			
Female	13 (39.4%)	11 (42.3%)						
ECOG PS								
0–1	9 (27.3%)	4 (15.4%)	2.062	0.555–7.661	0.280			
2	24 (72.7%)	22 (84.6%)						
Chemotherapy								
Monotherapy	11 (33.3%)	16 (61.5%)	3.200	1.096–9.343	0.033	2.948	0.910–9.553	0.072
Combination therapy	22 (66.7%)	10 (38.5%)						
TNM stage								
III	5 (15.2%)	5 (19.2%)	0.750	0.192–2.930	0.679			
IV	28 (84.8%)	21 (80.8%)						
Primary tumor location								
Head and neck	15 (45.5%)	16 (61.5%)	0.521	0.183–1.482	0.222			
Body and tail	18 (54.5%)	10 (38.5%)						
CA19-9 (U/ml)								
< 1000	27 (81.8%)	13 (50.0%)	4.500	1.394–14.528	0.012	3.703	1.056–12.988	0.041
≥ 1000	6 (18.2%)	13 (50.0%)						
Radiomics signature								
< 0.045	24 (72.7%)	11 (42.3%)	3.636	1.220-10.836	0.020	3.382	1.031–11.101	0.044
≥ 0.045	9 (27.3%)	15 (57.7%)						

### Correlations between clinicopathological characteristics and treatment effect

As shown in Table 3, in the univariate analysis, the non-progressive group was treated more by combination therapy than the progressive group (P = 0.033). The progressive group had a higher level of CA19-9 compared with the non-progressive group (P = 0.012). However, other factors including age, gender, ECOG PS, TNM stage, and primary tumor location, showed no significant differences between the two groups. Furthermore, multivariate logistic regression analysis showed RS and CA19-9 were independent predictive factors (P < 0.05), and a trend was found for chemotherapy (P = 0.072) between the two groups.

### Development and validation of the radiomics nomogram

**Fig. 3:**
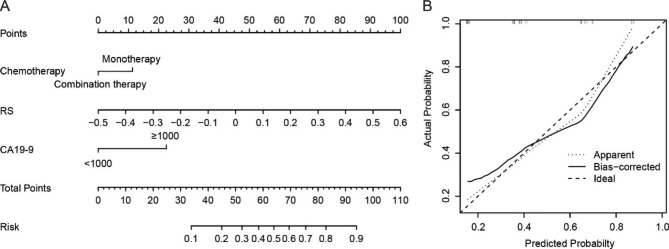
Developed the radiomics nomogram to predict the risk of disease progression after treatment with chemotherapy in a given patient (**A**). The calibration curves for the nomogram. The diagonal dotted line represents the perfect prediction of an ideal model. The apparent line represents the uncorrected performance of the nomogram, and the bias-corrected line represents the bias-corrected performance using bootstrapping with 1000 resamples (**B**)

A nomogram of the RS, CA19-9 and chemotherapy was constructed (Fig. 3A). The probability of high- or low-risk of disease progression after two cycles of chemotherapy was determined based on the total points of the radiomics nomogram. Furthermore, the calibration curve showed that the performance of the nomogram was similar to the ideal model and had good prediction capability (Fig. 3B). The nomogram achieved good discriminative ability in the training (C-index = 0.802) and validation (C-index = 0.920) cohorts. Comparably, the nomogram comprised of CA19-9 and chemotherapy only achieved moderate discriminative ability in the training (C-index = 0.705) and validation (C-index = 0.805) cohorts.

**Fig. 4:**
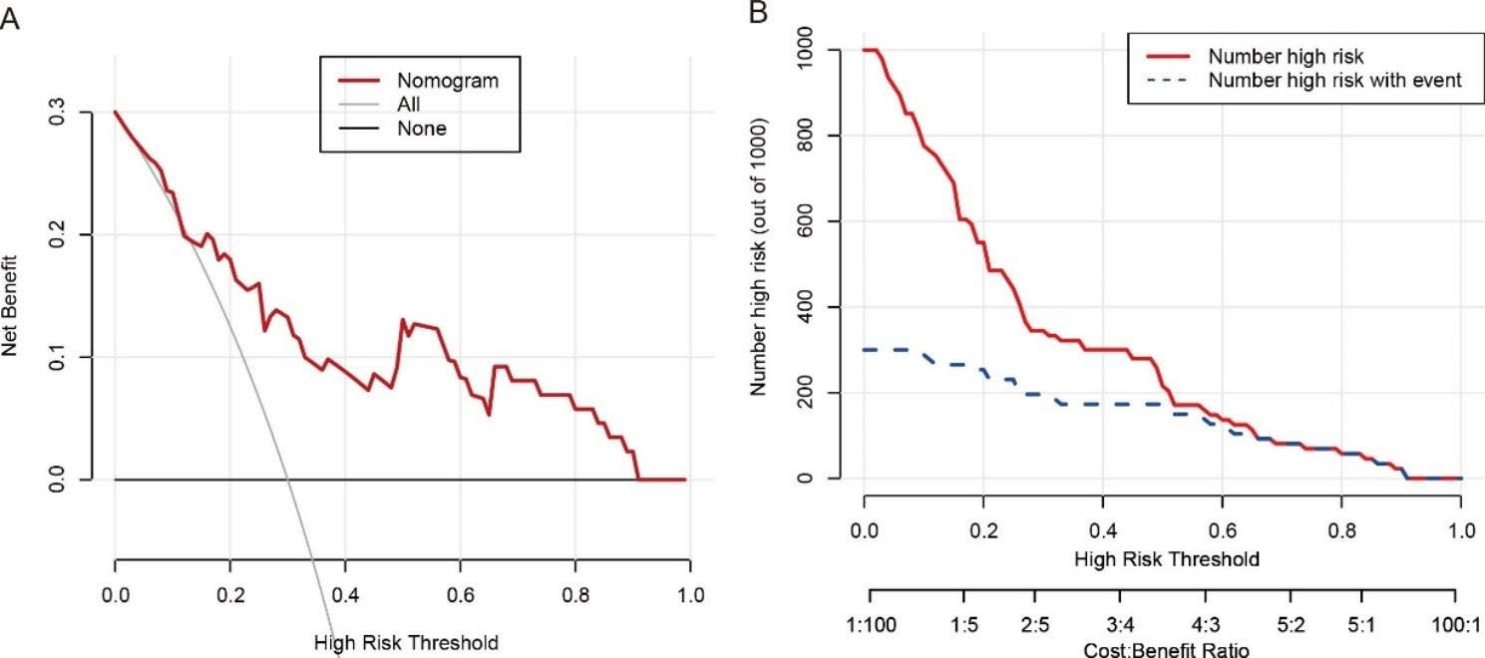
Decision curve analysis for the radiomics nomogram (**A**). The gray line shows the assumption that all patients had the risk of disease progression after treatment. The black line represents the assumption that no patients had the risk of disease progression after treatment. Clinical impact curve for the radiomics nomogram. The red curve represents the number of people classified by the nomogram as positive (high risk) at each threshold probability. The curve (Number high risk with event) is the number of true positives at each threshold probability (**B**)

The decision curve analysis (DCA) and clinical impact curve of the nomogram are shown in Fig. 4. The decision curves show that with a threshold probability > 0.4, using the radiomics nomogram to predict the efficacy of first-line therapy treatment response added more benefit than the scheme for all patients with a risk of disease progression after treatment or no patients with risk of disease progression after treatment.

## Discussion

In this study, RS was identified as a potential predictive biomarker for patients with APC treated with first-line chemotherapy. Furthermore, we integrated CA19-9, chemotherapy and RS to generate an innovative individualized radiomics model, in order to characterize PD responder and non-PD responder to first-line chemotherapy and determine the clinical application of the radiomics model in patients with APC.

Previous studies have assessed the predictive significance of radiomics features for the treatment response in various malignancies [16–18]. Several studies have reported that pre-treatment radiomics features are associated with therapeutic effects and survival after adjuvant chemotherapy or radiotherapy in patients with PDAC [19, 20]. For unresectable APC patients, Cheng et al. reported that the combination of pretreatment SD with tumor size achieved an early prediction of treatment with chemotherapy [21]. Salinas-Miranda et al. found that the CT texture feature of cluster tendency was a significant prognostic factor treated with chemotherapy [22]. However, most studies focused on patients’ survival after treatment, but not the response. In this study, we found that the final selected texture features, GLZLM_LZLGE, GLZLM_LGZE, and Energy could predict the early efficacy of first-line chemotherapy in patients with APC, and distinguish the PD responder from non-PD responders. The RS was an independent predictor of the early response to chemotherapy in the training cohort (p < 0.05).

In this study, the final selected texture features, GLZLM_LZLGE, GLZLM_LGZE, and Energy, used for the RS were significant predictors of chemotherapeutic effects. Except for energy, which belongs to the first-order feature, the other two were second-order texture features belonging to the GLZLM. The LZLGE and LGZE were based on GLZLM, representing the size of the homogeneous regions with the same gray level in three dimensions without a specific orientation. LZLGE shows the distribution of the long homogeneous zones with low gray levels [23]. LGZE represents the distribution of low gray-level zones [24]. Several studies found that tumor heterogeneity was correlated with a poor response to chemotherapy or chemoradiotherapy in patients with APC [11, 25]. The lower was GLZLM_LZLGE or GLZLM_LGZE, the more heterogeneous was the texture. It has been reported that pancreatic cancer cells induce fibrosis by increasing extracellular matrix synthesis, which may lead to chemoresistance [26]. Thus, our results could be interpreted that the higher value of GLZLM_LZLGE or GLZLM_LGZE, the more homogeneous the tumor was, indicating less related fibrosis and higher sensitivity to chemotherapy. Energy reflects the homogeneity of gray distribution and the roughness of texture. The more homogeneous the image, the higher the energy [27]. However, it has also been reported that homogenous texture may indicate higher cellular density, reducing the amount of drug delivered to the tumor, leading to chemotherapy resistance [28]. Thus, our results could be interpreted that the higher value of Energy, the higher cellular density the tumor had, indicating lower sensitivity to chemotherapy.

Several studies found that the preoperative elevated CA19-9 level was a significant independent factor for predicting poor prognosis in patients with PDAC [29–32]. And other studies also found the baseline CA19-9 level was an independent risk factor for prognosis in patients with PDAC receiving Gemcitabine together with nab-paclitaxel [33, 34]. Our result was consistent with previous findings. Our study found that the CA19-9 level was an independent predictive marker in early chemotherapeutic effects for APC patients. The multivariate logistic regression analysis showed that the chemotherapy did not demonstrate enough predictive ability (P = 0.072), which made it seem unnecessary for inclusion in the model. However, several studies found that chemotherapy was an independent prognostic factor in the outcome of advanced pancreatic cancer patients [35, 36]. Moreover, several studies found that nab-paclitaxel plus gemcitabine combination therapy significantly improved survival than gemcitabine monotherapy in patients with APC [37, 38]. The lack of statistical significance of chemotherapy in the validation cohort may be due to the relatively small sample size and confounding by other factors [39]. The nomogram incorporating RS, CA19-9 and chemotherapy showed better discriminative ability in the training (C-index = 0.802) and validation (C-index = 0.920) cohorts than the one incorporating only CA19-9 and chemotherapy.

Based on the total points of the nomogram, a high- or a low-risk probability of disease progression after two cycles of chemotherapy treatment was determined. The nomogram calibration curve showed good agreement between the predicted and observed outcomes. The individualized prediction of treatment effect using the nomogram based on CT texture features and routine noninvasive tests is necessary for clinicians to make more precise decisions, which aligns with the trend of personalized medicine. Specifically, if a patient is determined to have a high-risk probability of disease progression according to the nomogram, more aggressive treatment is prone to be given. Patients with APC will benefit from this novel approach by providing risk stratification and decision support. Moreover, DCA and clinical impact curve were used to ensure that the nomogram it had good clinical utility. These findings indicated that it was an effective way to predict the early treatment response for APC patients by using CT texture feature-based nomogram.

Our study also has some limitations. First, the sample size is relatively small. We will continue to expand the sample size and conduct a multi-center study to confirm our findings. Second, manual segmentation was adopted in this study, which could introduce subjective bias to a certain extent. Third, our study only analyzed the images of patients from the arterial phase; although the lesions are well shown in the arterial phase, we still need to explore the noncontract phase or portal vein phase to enrich the results we investigated. Furthermore, our study is based on 2D images, and the lesion may not be fully reflected in the largest cross-section of the tumor, so we will use 3D images to extract the entire tumor in the future.

In conclusion, we developed a pre-treatment CT-based radiomics nomogram to predict the early efficacy of first-line chemotherapy in patients with APC, distinguish the PD responder from non-PD responder to first-line chemotherapy treatment. Our initial results showed that the nomogram, including RS, CA19-9 and chemotherapy provide a promising way to predict early chemotherapeutic effects for APC patients.

## Data Availability

The datasets generated and analyzed during the current study are not publicly available due to the fact that they constitute an excerpt of research in progress but are available from the corresponding author on reasonable request.
